# Supplementary material for: Transition from AFM Spin Canting to Spin Glass–AFM Exchange as Particle Size Decreases in LaFeO3
Source: Nanomaterials (Basel). 2023 May 17;13(10):1657. doi: 10.3390/nano13101657 (PMC10224450; doi:10.3390/nano13101657)
Supplement: Supplementary file 1 [file nanomaterials-13-01657-s001.zip › nanomaterials-2396236-supplementary.pdf]

Transition from AFM Spin Canting to Spin Glass–AFM Exchange as Particle Size Decreases in LaFeO3

S1. Compositional analysis

Table S1: XEDS analyses in LF-800 sample.

Fe Atomic (%)	La Atomic (%)	Stoichiometric Composition
42.68	57.32	$La_{1.15}Fe_{0.85}O_3$
45.98	54.02	$La_{1.08}Fe_{0.92}O_3$
45.04	54.96	$La_{1.10}Fe_{0.90}O_3$
44.78	55.22	$La_{1.10}Fe_{0.90}O_3$

48.12	51.88	$La_{1.04}Fe_{0.96}O_3$
43.98	56.02	$La_{1.12}Fe_{0.88}O_3$
45.10	54.90	$La_{1.10}Fe_{0.90}O_3$
45.21	54.79	$La_{1.10}Fe_{0.90}O_3$
44.55	55.45	$La_{1.11}Fe_{0.89}O_3$
44.87	55.13	$La_{1.10}Fe_{0.90}O_3$
45.11	54.89	$La_{1.10}Fe_{0.90}O_3$
46.25	53.75	$La_{1.08}Fe_{0.92}O_3$
47.09	52.91	$La_{1.06}Fe_{0.94}O_3$
45.51	54.49	$La_{1.09}Fe_{0.91}O_3$
49.52	50.48	$La_{1.01}Fe_{0.99}O_3$
44.80	55.20	$La_{1.10}Fe_{0.90}O_3$
46.36	53.64	$La_{1.07}Fe_{0.93}O_3$
45.84	54.16	$La_{1.08}Fe_{0.92}O_3$
45.58	54.42	$La_{1.09}Fe_{0.91}O_3$
43.97	56.03	$La_{1.12}Fe_{0.88}O_3$
47.48	52.52	$La_{1.05}Fe_{0.95}O_3$
44.25	55.75	$La_{1.12}Fe_{0.88}O_3$
48.26	51.74	$La_{1.04}Fe_{0.96}O_3$
43.51	56.49	$La_{1.13}Fe_{0.87}O_3$
		**$La_{1.09}Fe_{0.91}O_3$**

S2. XRD Diffraction

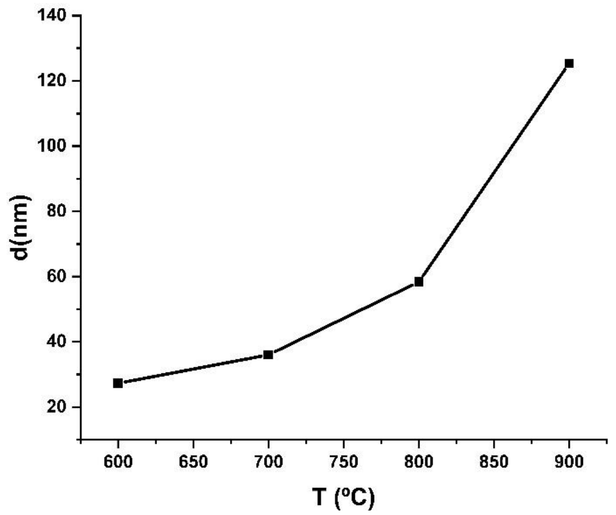

Figure S1: Mean crystallite size (nm) calculated by Scherrer's equation for all sol-gel samples LF-600, LF-700, LF-800, and LF-900.

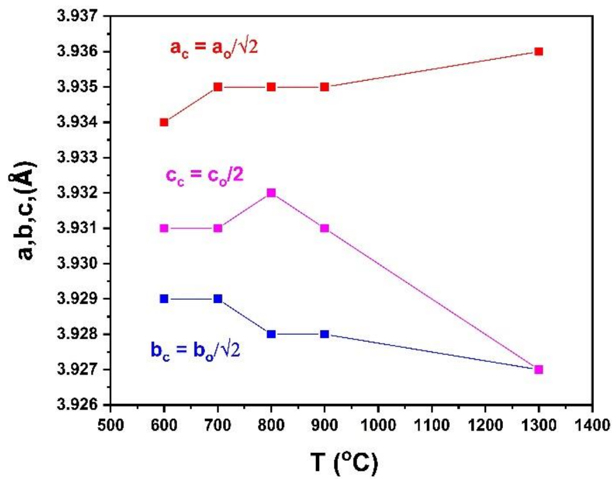

Figure S2: The variation values of cell parameters a, b, and c as a function of annealing temperature.

S3. High resolution microscopy.

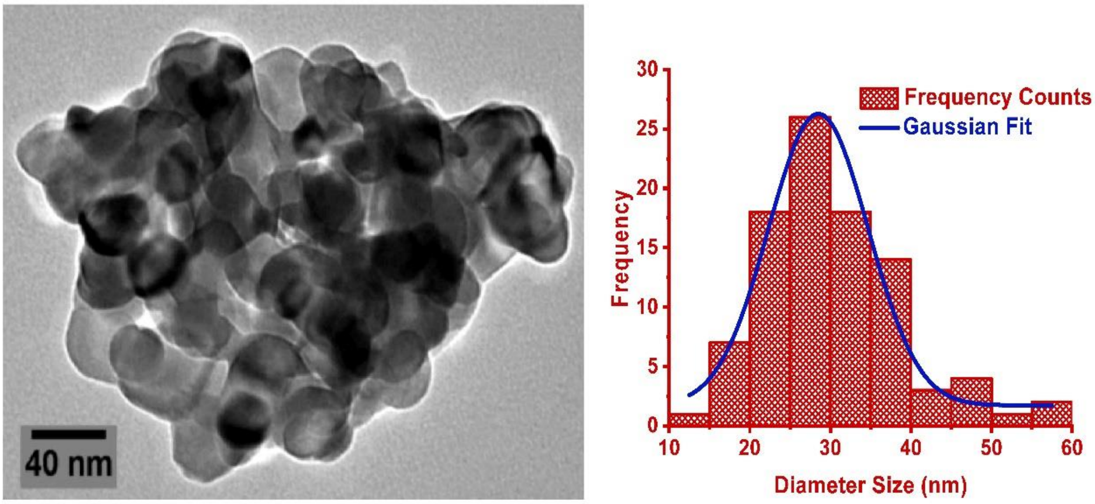

LF-700

Figure S3: (Left) LF-700 TEM image. (Right) Distribution of the nanoparticle diameters with Gaussian fit.

S4. Magnetic properties

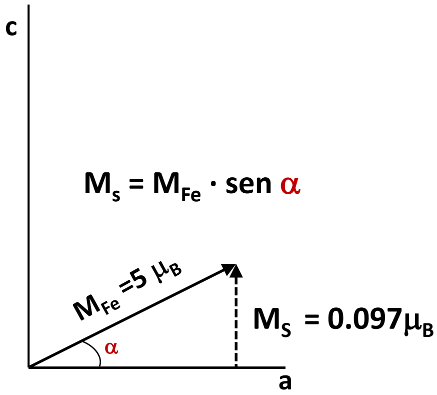

Figure S4: Spin canting angle.
